# Development and Evaluation of the Implementation of Guidelines for Healthier Canteens in Dutch Secondary Schools: Study Protocol of a Quasi-Experimental Trial

**DOI:** 10.3389/fpubh.2019.00254

**Published:** 2019-09-06

**Authors:** Irma J. Evenhuis, Ellis L. Vyth, Lydian Veldhuis, Jacob C. Seidell, Carry M. Renders

**Affiliations:** ^1^Department of Health Sciences, Faculty of Sciences, Amsterdam Public Health Research Institute, Vrije Universiteit Amsterdam, Amsterdam, Netherlands; ^2^Netherlands Nutrition Centre, The Hague, Netherlands

**Keywords:** schools, nutrition, canteen, adolescents, implementation

## Abstract

**Introduction:** To encourage healthier food/drink choices, the “Guidelines for Healthier Canteens” were developed by the Netherlands Nutrition Centre. This paper describes (1) how we developed a plan to support implementation of the “Guidelines for Healthier Canteens” in Dutch secondary schools, and (2) how we will evaluate this plan on process and effect level.

**Materials and Methods:** The implementation plan (consisting of several tools) was developed in cooperation with stakeholders. Barriers/facilitators to implement the guidelines were identified by 14 interviews and prioritized during one expert meeting. Thereafter, these barriers were translated into implementation tools using behavioral change methods and implementation strategies. The implementation plan consists of the tools: tailored advice provided via an advisory meeting and report, based on a questionnaire about the stakeholders'/school's context and the “Canteen Scan,” an online tool to assess the product availability and accessibility; communication materials; an online community; newsletters; a factsheet with students' wishes/needs. This implementation plan will be evaluated on process and effect in a 6-month quasi-experimental controlled design with 10 intervention and 10 matched control schools. Process outcomes will be measured: (1) factors affecting implementation and (2) the quality of implementation, both collected via a questionnaire among involved stakeholders. Effect outcomes will be collected pre/post-intervention with: (1) self-reported purchase behavior among around 100 students per school; (2) the “health level” of the school canteen. Linear and linear/logistic two-level regression analyses will be performed.

**Discussion:** The implementation tools are developed by combining a theory and practice-based approach, with input from different stakeholders. If these tools are evaluated positive, it will support schools/stakeholders to create a healthier school canteen.

**Trial Registration:** Dutch Trial register no.: NTR5922, date of registration June 20, 2016; METC no.: 2015.331; EMGO+ project number: WC2015-008.

## Introduction

Prevention of overweight and obesity during childhood is important because of the high prevalence worldwide and associated short and long-term physical, social and mental health problems (1–4). Although prevention should start in early life, adolescence is also a critical period for prevention, because adolescents start to deal with more responsibilities, and develop their own identity and habits in eating behavior, which may persist in later life (5, 6). To promote healthy dietary behavior, it is important to change the food environment to stimulate individuals toward healthier food choices (7–10). For adolescents, schools are a key setting to encourage healthy eating as schools have a pedagogical task and a large reach, and adolescents spend a lot of time there (10, 11). Although schools are increasingly aware of their role in obesity prevention and the need for a healthier school canteen, there is room for improvement (12–14). Schools often experience barriers to implement a healthier school canteen and need support to implement and continue actions regarding a healthier school canteen (14, 15). Hence, improvements in the canteen like removing the marketing of less healthy products and increasing the offer of healthier food and drinks in vending machines remain difficult (12, 13).

Decreasing the availability of low-nutrient, energy-dense foods/beverages in comparison to high-nutrient, low energy foods/beverages in the school canteen and vending machines, and formulating relevant school food policy, are examples of promising strategies to change the food environment and reduce consumption of low nutritious foods, and increase purchases of favorable foods/beverages (16–19). The Dutch Ministry of Health, Welfare and Sport has set a policy target to increase the number of schools with a healthier canteen (20). The Netherlands, has around 1,500 secondary schools, which offer different educational levels for youth between the ages of 11 to approximately 18 years. Most schools offer food or drinks for sale as substitute to the food/drinks students bring from home. In 2014, the Netherlands Nutrition Centre developed the “Guidelines for Healthier Canteens” in consultation with future users and experts in the field of food and behavior change (21). These guidelines are based on studies which investigated influences on making choices, the Dutch Nutritional guidelines “The Wheel of Five,” and experiences with the “Healthy School Canteen” programme (22, 23). According to the “Guidelines for Healthier Canteens” school canteens should offer a majority of healthier products. Healthier products are defined as foods and drinks that are included in the Dutch “Wheel of Five,” such as whole wheat bread, fruit and vegetables, and products that are not included, but contain a limited amount of calories, saturated fat, and sodium (22). In addition, the canteen should promote healthier products by applying “accessibility criteria,” such as placing the healthier products at the most eye-catching spots and attractive presentation of fruit and vegetables. Further, drinking water should be encouraged and in its written policy, the school should state that their canteen meets the guidelines (21).

Stakeholders need support to implement the guidelines in their school (15, 26, 27). Such an implementation support plan will be better aligned to the needs of practice, and thereby more feasible, if the needs and wishes of stakeholders are taken into account (9, 28, 29). Therefore, during the development and evaluation stage, collaboration with these stakeholders is recommended (28, 29). It is also recommended to apply theory, such as the use of a structural framework for the development and evaluation of the implementation plan, the use of behavior change models to translate the need of practice into implementation strategies and the use of a combination of implementation tools (30, 31). The collaboration with practice in combination with the use of theory will increase the likelihood of a feasible and effective implementation. To succeed over time, implementation of new guidelines should allow adaptations to local circumstances but, nonetheless, be conducted with rigor and consistency. This article describes: (1) how we developed a plan to support implementation of canteen guidelines in Dutch secondary schools; and (2) how we will evaluate this implementation plan on process and effect level. The process will be evaluated on factors affecting implementation perceived by stakeholders and the quality of implementation. The effect will be evaluated by determining changes in the health level of canteens and in the self-reported purchase behavior of adolescents.

The input of practice during the development and evaluation of our implementation plan will give insights to researchers about working elements. We hypothesize that this approach will increase future uptake and effect of the implementation plan. With our implementation plan we aim to facilitate the process to create a healthier school canteen, and thereby to stimulate Dutch adolescents to purchase healthier foods and beverages during school time.

## Methods

**Figure 1:**
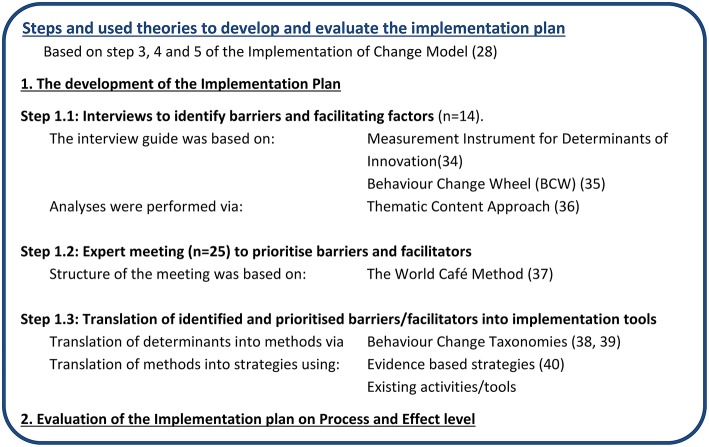
Steps and used theories to develop the implementation plan.

Many approaches to support the development and evaluation of implementation interventions exist and have corresponding steps (30–32). In this study the “Grol and Wensing Implementation of Change Model” (2006, updated in 2016) was used to develop and evaluate the implementation plan to disseminate the Guidelines for Healthier Canteens in secondary schools (30). A strength of this model is that it combines several approaches and has been improved over time. It consists of six steps from developing a proposal for change when new guidelines are developed to continuous evaluation and adaptation of the implementation plan. The first two steps are not applicable as the guidelines already exist. The last step falls outside the scope of this research but will be aimed to perform in the future. Hence, this paper describes the application of the three middle steps: (3) the need assessment of the target group and setting, (4) the selection of corresponding implementation strategies, and (5) the development, testing, and executing of the implementation plan. In the selection of implementation strategies, characteristics of the Intervention Mapping approach are used (31). We divided our study into two phases: first the development, which has already been performed and second the evaluation of the implementation plan. These phases and a timeline are presented in Figure 1 and explained below. To report this study design, the SPIRIT 2013 Statement was used, if applicable (33). As a full description of an implementation plan makes it possible to use it in practice, to compare results and to enhance reproducibility (34), this article explains how we developed and will evaluate the implementation plan, while a separate article will describe the content of the implementation plan. Namely, by describing the factors aimed to change with the plan, the behavioral change methods, implementation strategies and an explanation of the implementation tools.

### Development of the Implementation Plan

We developed the implementation plan in three steps. We started with interviews, to gather information on barriers and facilitators regarding a healthier school canteen according to relevant stakeholders of policy and practice. Next, experts from research, policy, and practice prioritized the identified barriers and facilitators and came up with solutions. Subsequently, behavior change methods and implementation strategies were assigned and translated into implementation tools, corresponding to the most important barriers/facilitators identified.

#### Interviews to Identify Barriers and Facilitating Factors

##### Design, participants, data collection

The aim of this qualitative study was to identify barriers and facilitators, both experienced and expected, by users and stakeholders of the school canteen due to the Guidelines for Healthier Canteens. Furthermore, they came up with possible solutions for the perceived barriers. These insights helped to develop an intervention that was aligned to the need of practice and their daily practice. Semi-structured interviews were conducted among purposive sampled users and stakeholders on organization level. Users were defined as persons responsible for the school canteen and who will use the Guidelines for Healthier Canteens in the future (e.g., a schools' facility manager, a coordinator, or a caterer). In addition, school canteen advisors were included as “users.” They are dieticians of the Netherlands Nutrition Centre who visit, advise and support Dutch schools and caterers aiming to achieve healthier school canteens. Stakeholders on organization level were the managers of schools and caterers.

Participants were recruited via the school canteen advisors of the Netherlands Nutrition Centre. Fifteen stakeholders and users were invited for the interviews by e-mail or telephone; one stakeholder was unable to attend because of organizational changes. Experiences of school canteen advisors of the past years showed that some organizations just started, while others were already experienced to create a healthier canteen. To get more insight into these differences, we included participants spread among the Rogers' diffusion of innovation theory (35). The included participants were spread among innovators (*n* = 5), the majority (*n* = 7), and laggards (*n* = 2). The Guidelines for Healthier Canteens were sent to the participants and informed consent was signed before the interview. A researcher (IE) trained in qualitative interview methods conducted the interviews and a second researcher was present to make notes. After the interviews, a member check was conducted. As the last interviews did not reveal any new information, we concluded that data-saturation was reached.

##### Interview topics

The fourteen interviews were structured around open-ended questions. The topic list was compiled using the most important determinants of the Measurement Instrument for Determinants of Innovation (MIDI) and the Behavior Change Wheel (BCW) (24, 25). The MIDI includes 29 determinants of innovation categorized into determinants of users, organization, innovation, and social political environment. The BCW describes capability, opportunity and motivation (all of which interact with each other) as most important determinants that are needed for behavioral change. The topic list consisted of the main-topics: context, experience, opinion about the guidelines, desired support and solutions and completion. After each interview the topic list was optimized, based on experience with the earlier interviews.

##### Data analysis

All interviews were audio-taped and transcribed verbatim. The thematic content approach was used for data collection and data analysis (36). Three steps were undertaken to analyse the interviews; open, axial and selective coding. Coding process was performed by two researchers, in alignment with each other and with a third researcher (IE). Thereafter, results were discussed with the project team.

#### Expert Meeting to Prioritize Barriers and Facilitators

##### Design and participants

As many factors were identified from the interviews, it was needed to discuss together with different stakeholders which factors should be affected at least by the intervention. To prioritize the identified barriers and facilitators an expert meeting was organized with attendees from research, policy and practice. A total of 30 experts were invited, e.g., managers at school/caterers, health promoters from the Community Health Services and the Healthy School Program, school canteen advisors, and researchers in the field of implementation, nutrition and behavior. A total of 25 experts participated, divided over research (*n* = 10), policy (*n* = 4), and practice (*n* = 11).

##### Data collection

The expert meeting consisted of two parts. First, the 41 barriers and facilitators retrieved from the interviews were prioritized to create focus which factors needed to be changed with the implementation plan. Each participant first ranked all barriers and facilitators individually, thereafter plenary all factors were discussed and consensus about the prioritization was reached. Second, solutions to strengthen facilitators and reduce barriers were identified and discussed in in six subgroups, based on the World Café Method (37). To provide participants already with ideas, all groups received a list with current implementation tools, and solutions suggested by participants of the interviews. The results of the expert meeting were multiple ideas to influence the highest-ranked facilitating and impeding factors.

#### Translation of Identified and Prioritized Barriers/Facilitators Into Implementation Tools

The prioritized barriers and facilitating factors were translated into corresponding implementation tools through behavior change methods (techniques) and implementation strategies (38–40). This theory based translation was needed as it is important to choose strategies that—from a theoretical perspective—are likely to change the prioritized factors. The implementation plan consists of a mix of activities and tools, so called implementation tools, aiming to change the crucial and most important impeding and facilitating factors that affects implementation (30). The choices made for implementation tools were grounded in evidence-based theory, existing (and previously used) tools and activities of the Netherlands Nutrition Centre, and by balancing the expected effect and investment (financial, time-consuming, effort, commotion) (38, 39). The tools were developed in collaboration with the project team, and organizations which will support implementation in the future [e.g., the Netherlands Nutrition Centre, the Amsterdam Community Health Service, and “Youth on a Healthy Weight (JOGG)”].

### Evaluation of the Implementation Plan on Process and Effect Level

#### Setting and Study Design

**Figure 2:**
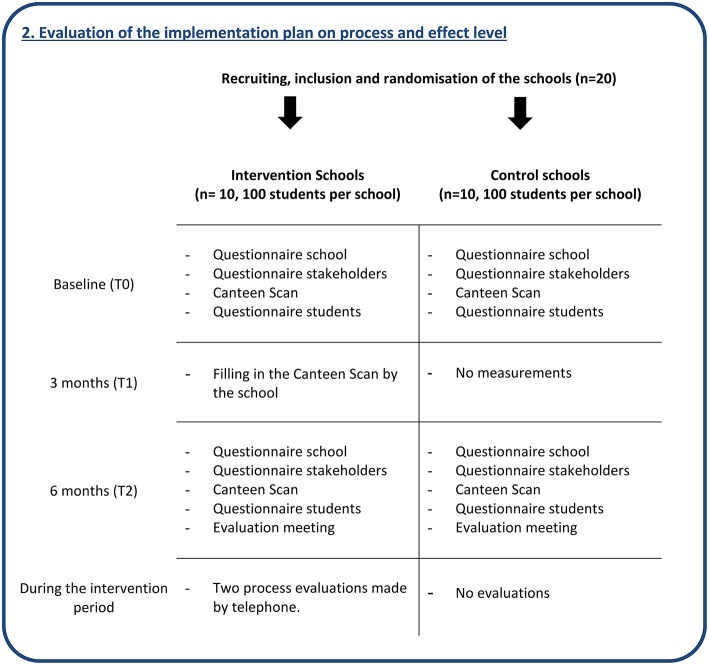
Evaluation of the implementation plan on process and effect level.

To evaluate both the process and effect of the developed implementation plan, a 6-month quasi-experimental controlled design will be used with 10 intervention and 10 matched control schools (see Figure 2). The included schools will have a variety of characteristics, so the results can be translated to other Dutch schools. Control schools will be matched by the main characteristics: how the catering is provided (i.e., by a catering company, or the school itself), school size (<1,000 and ≥1,000 students), level of secondary education (vocational, senior general, and pre-university), availability of (many) shops near the school, and whether or not the school has a policy for students to stay on the schoolyard during breaks. Intervention schools will receive the developed implementation plan to support implementation of the Guidelines for Healthier Canteens, whereas the control schools will receive the guidelines only. Control schools will receive these guidelines in a short meeting and on paper after the baseline measurements. After the intervention period, control schools will receive the intervention. This quasi-experimental study will be carried out according to: (1) the project application (Nr: 50-53100-98-043, date: 2 December 2014) approved by funding organization ZonMw, (2) the study protocol approved by the VU University Medical Centre (WC2015-008 and 2015.331), and (3) registration in the Dutch Trial Register (NTR5922).

#### Study Population and Recruitment

##### Schools

We will recruit schools that are situated in the western and middle part of the Netherlands, via the Netherlands Nutrition Centre and caterers by email and telephone. The inclusion criteria are: (a) presence of a canteen, (b) willingness to make their school canteen healthier, (c) and willingness to provide time and space for the investigators to measure outcomes in students, employees, and canteen workers. The exclusion criteria are: (a) the school had already started to implement the recent developed Guidelines for Healthier Canteens, and (b) in 2015, the school canteen had already been advised about how to reach a healthier canteen, by school canteen advisors. After 6 months of participation in all measurements, all schools will receive a small financial incentive.

##### Stakeholders

In the participating schools, all stakeholders involved in implementing a healthier school canteen will be asked to fill in questionnaires at baseline and after the intervention. These stakeholders will be identified by our contact of the school. The number of stakeholders and their function will differ per school, due to organizational differences between schools. Involved stakeholders may include: teachers, students, representatives of the school board/school canteen, students, and health promoters of the Community Health Service.

##### Students

In each of the participating schools, 100 second or third-year (aged 13–15 years) students will be included. Therefore, approximately four second-year classes will be invited to participate, reflecting the education levels offered at the school. Students will be asked to fill in a questionnaire, at baseline and after the intervention. Two weeks prior to the questionnaires, parents, and students will receive an information letter, and the option to decline participation. Per school, four vouchers of €25 (for an online goods shop) will be raffled off among all participating students.

#### Intervention

The implementation plan, consisting of various implementation tools, was developed as described before. Some existing tools were adapted and others were newly developed in collaboration with stakeholders from research, policy and practice. This resulted in a mix of implementation tools (Table 1): a questionnaire to gain insight in stakeholders' and schools' specific context; the Canteen Scan (an online tool that provides insight and advices regarding the availability and accessibility of food and drink products in their canteen); an advisory meeting and written report in which stakeholders receive tailored advice; communication materials; an online community; newsletters; and a factsheet with students' needs and wishes. During the intervention all schools will be encouraged to involve their students in the process to change their canteen. The implementation tools will be provided by school canteen advisors of the Netherlands Nutrition Centre, in collaboration with the Vrije Universiteit Amsterdam. Within our research, the advisors will use the developed implementation tools to support the intervention schools.

**Table 1:** Description of the tools for implementation of the guidelines for healthier canteens.

**Implementation tool**	**Action and targets**	**Target group**	**Period**
1. Insight in the current situation			
1.1: Questionnaire school	The results of the online questionnaire to assess the characteristics of the school (25, 41) are given back to the stakeholders	Coordinator of the school, all involved stakeholders	Before/during the advisory meeting
1.2: Questionnaire stakeholders	The results of the online questionnaire to assess stakeholders' characteristics, individual and environmental determinants (25, 41) are given back to the stakeholders	All involved stakeholders	Before/during the advisory meeting
1.3: “Canteen Scan”	An online tool that provides insight into and directions for improvement of availability and accessibility of food and drink products in canteens (42)	Performed by a school canteen advisor of the Netherlands Nutrition Centre. Results and advise are given to all involved stakeholders	Before the advisory meeting
	To create ownership and insight into the changes so far, the school receives information to fill out the Canteen Scan by themselves if they wanted	Performed by the school coordinator	After 3 months
1.4: Advisory meeting and report	In one advisory meeting per school, all involved stakeholders are advised about how to improve the canteen by a school canteen advisor of the Netherlands Nutrition Centre. Based on the aims of the school and the points of attention, identified with the two questionnaires and the Canteen Scan a concrete action plan will be developed during the meeting. As this action plan is created together, ownership and collaboration will be increased. After the meeting, a written report based on this meeting will be distributed by email	All involved stakeholders	At the start of implementation
2. Communication materials	A brochure about the Guidelines for Healthier Canteens, an overview of the steps to take, a personalized poster, a banner for the schools' website. To create motivation and increase and apply knowledge. Content: information, examples of healthier products, how to place products, and healthier canteens	Coordinator of the school, who will be asked to share this with other stakeholders	At the start and halfway of implementation
3. Online community	A closed Facebook community for stakeholders to share their experiences, ask questions and support each other	All stakeholders	Continuous
4. Digital newsletter	A regularly newsletter send by email, consisting of information and examples regarding the healthier school canteen	All stakeholders	Every 6-weeks
5. Students' factsheet	A summary of their students' wishes and needs regarding a healthier school canteen, to receive insight into the opinion of their students and how their students want to be involved	Coordinator of the school, who will be asked to share this with other stakeholders	Once, 2–4 weeks after the start

#### Outcomes

##### Process evaluation

All stakeholders involved in implementing the healthier school canteen will be asked to fill in an online questionnaire pre and post-intervention. Demographics will be measured of stakeholders (e.g., age, gender) and school (e.g., offered education level, number of students).

The first process evaluation outcomes are perceived individual factors of the stakeholders and environmental factors that can affect the implementation process. Pre and post-intervention, these individual factors (e.g., knowledge, self-efficacy, and attitude regarding a healthier school canteen), as well as environmental factors affecting implementation (e.g., need for support, innovation, and organization) will be measured, based on the validated Theoretical Domain Framework questionnaire (41) and the Measurement Instrument for Determinants of Innovations [(25); Table 2].

**Table 2:** Overview of the process and effect evaluation measures, assessed at stakeholders, students, or canteens.

**Measure**	**Response options**	**Concepts**	**Example**
**Process evaluation measures^a^**			
**Questionnaire for stakeholders (measured at T0 and T2)**			
Demographics	Frequencies, multiple choice, Open question	Age, gender, function, offered education level at school, number of students	What is your main function at work?
Individual factors affecting implementation of the healthier school canteen	5-point Likert scale	Knowledge, attitude, self-efficacy, social influence, motivation, routine, intention, skills, professional role, behavioral regulation	I have enough knowledge to create a healthier school canteen.
Environmental factors affecting implementation of the healthier school canteen	5-point Likert scale	Need for support, Innovation, organization, current behavior for school canteen	I need (more) support to adequately perform my activities for a healthier school canteen
Overall evaluation of the implementation process^b^	Open-ended question	Positive experiences, negative experiences, suggestions for improvements	What suggestions would you give to a school that is just starting to create a healthier school canteen?
Quality of implementation^a^^,^ ^b^^,^ ^c^^,^ ^d^	Dichotomy and 5-point Likert scale	Dose delivered, dose received, satisfaction	Have you read/used the *[implementation tool]*? (yes/no) How satisfied are you with the *[implementation tool]*? (1–10)
**Effect evaluation measures**			
**Questionnaire purchase behavior and determinants of purchase behavior of students (measured at T0 and T2)**			
Demographics	Frequencies, multiple choice	Age, gender, education level	What is your current age?
Purchase behavior of foods and drinks	Frequencies	In school at the counter In school at vending machines	How often per week do you buy fruits at the school counter?
Behavioral determinants of healthy purchase behavior at school	5-point Likert scale	Attitude Perceived behavioral control Subjective norm	Next month, I intend to buy healthier products in the school canteen
Environmental determinants of healthy eating behavior during school time	Multiple choice	Breakfast behavior Money spending at school Food and drinks brought from home Food and drinks bought outside school	I bring foods to school (0–>5) times a week
**Canteen Scan (measured at T0 and T2)**			
Health level of the canteen (availability and accessibility of healthier food and drinks)	Multiple choice	Basic conditions for all canteens	Encourages the school canteen people to drink water? (i.e., by water tap)
	Open Question	Percentage of available healthier food and drinks on display	Please enter all products on display (at the counter, in display cases and on racks) in the school canteen
	Open Question	Percentage of available healthier food and drinks in vending machines	Please enter all products in the vending machine
	Multiple choice	The canteen's accessibility criteria (to motivate people to select a healthier option)	Does the school canteen present fruit or vegetables in an attractive manner?

a *Asked for each implementation component*.

b *Only measured at T2*.

c *Only measured by the stakeholders of the intervention schools*.

d *Also measured by logging the use digital*.

The second process evaluation outcome is the quality of implementation. After 6 months, all stakeholders in the intervention group will be asked to evaluate the quality of each implementation tool. With an online questionnaire, quantitative process evaluation measures derived from the methodology of Saunders et al. (43) and Steckler and Linnan (44) will be measured. Fidelity will be measured with dose delivered and dose received. In addition, satisfaction will be measured. *Dose delivered:* Number of stakeholders to whom the tool was provided by the school canteen advisors. *Dose received:* Number of stakeholders who received and used the tool. *Satisfaction:* Participant's satisfaction with each tool. Additionally, objective data collection will be conducted by digitally logging the delivery and use of each online implementation tool. Moreover, after the intervention via open-ended questions in the questionnaire and during an evaluation meeting, all stakeholders will be asked to: explain their satisfaction score; give a short evaluation per implementation tool; give their positive and negative experiences overall; and to give their suggestions for improvements (qualitative data).

##### Effect evaluation

The effectiveness of the implementation process will be evaluated by measuring at baseline and at follow-up after 6 months via (1) the self-reported purchase behavior of students, and (2) the “health level” of the school canteen (Table 2).

The questionnaire to assess the primary outcome self-reported purchase behavior of students, the behavioral determinants of purchase behavior (Perceived behavioral control, attitude, and subjective norm of healthy eating in school) and the environmental determinants (like food brought from home, purchases during but outside school) is derived from existing validated Dutch questionnaires (45–49). The frequency of food/beverage purchases per week in the school canteen/vending machines of products that are the “healthier products” and products which should be consumed only occasionally, will be asked (21, 50). The questionnaire will be reviewed and discussed on face validity and content validity by all project members involved. Thereafter, it will be pretested by respondents of the same age as the target group using the cognitive interview method think-aloud (51). The aim of this pretest is to get insight into respondents' comprehensibility and the length of the questionnaire, to be able to adapt questions if needed (51). The questionnaire will be administered digitally in a classroom setting in the presence of a teacher or researcher.

The secondary outcome “health level” of the school canteen will be measured with the online tool, “the Canteen Scan.” This tool was developed and improved and improved in an iterative process through a collaboration of researchers, professionals, schools, caterers, and experts on nutrition and health behavior, and tested on its validity and inter-rated reliability (42, 52). The Canteen Scan checks to what extent a canteen meets the Guidelines for Healthier Canteens and subsequently provides tailored advice for improvements. The three parts of the guidelines can be entered in this tool: (1) a set of basic conditions for all canteens, (2) the food and drink available on display and in vending machines, and (3) the accessibility of healthier food and drink products (21, 42). Subsequently, the school canteen's current overall level (silver or gold), and a level for all three individual parts (in percentages) is indicated. Consequently, the health level of the canteen can be defined as: the available basic conditions, the available healthier food and drinks and meeting the accessibility criteria in the school canteen. The Canteen Scan will be filled out in all intervention and control schools by a school canteen advisor. Intervention schools will receive the outcome and feedback as part of the intervention. On the contrary, the control schools will not receive the results or feedback from the Canteen Scan.

#### Sample Size

The power calculation was based on the primary outcome, i.e., the self-reported purchase behavior of healthier products per week. In this calculation we included an 80% power and a 5% significance level (53). To detect a 10% difference in the proportion of purchasing healthier vs. unhealthier products per week (dichotomous variable) between the intervention and control group, with the expected multi-level structure between schools (correlation of 0.05 between schools), and to obtain sufficient power (80%), we calculated that 1,505 students spread among 10 intervention and 10 control schools are needed. The increase of 10% in purchase behavior of healthier products is based on results of comparable studies in schools (54). Consequently, we aimed to recruit 20 schools and 100 students per school, based on an expected dropout rate of 10% (55).

#### Statistical Analysis

##### Process evaluation

To test for differences in factors affecting implementation perceived by stakeholders (dependent variable) between the intervention and control group (independent variable) after the intervention (6 months), linear two-level regression analysis will be used. The used levels will be: stakeholders (level 1) and schools (level 2) and we will adjust for baseline measurements. This analysis will be performed for each individual (e.g., knowledge, attitude, and self-efficacy) and environmental factor (e.g., need for support, innovation). When these analyses show no significant difference between school variance, a linear regression analysis will be performed (53). We hypothesize that the stakeholders in the intervention group will positively change their perceived factors due to the support in implementation.

To investigate the quality of implementation quantitatively (dose delivered, dose received, and satisfaction) of each implementation tool, descriptive statistics will be used. This information will be complemented by qualitative data about the overall experiences of stakeholders. This data will be analyzed in three rounds, following the thematic content approach (36). First, answers will be labeled with descriptive codes. Second, the codes will be split or merged and interpretative codes will be created. Third, codes will be compared and overarching themes defined.

##### Effect evaluation

After the intervention, differences in the primary outcome “purchase behavior” of students (dependent variable) between the intervention and control group (independent variable) will be analyzed with two-level regression analysis (intention-to-treat). Here, we will correct for correlations of students (level 1) nested within schools (level 2). We will adjust for confounders related to students (e.g., groups of sociodemographic characteristics, behavioral determinants, and environmental determinants). In addition, the moderation effect of gender will be taken into account by stratifying the analyses, based on literature (56). We hypothesize that students in the intervention group will achieve a healthier purchase behavior.

After the intervention period, differences in the secondary outcome “health level” of the canteen between the intervention and control schools will be investigated with descriptive statistics. Thereafter, to gain insight into the effect of the health level of canteens and purchase behavior of students, we will include the health level of canteens in a per protocol analysis. This model will be built similar as the explained intention-to-treat analysis. All information is being gathered with rigor, so these analyses will show which factors make a difference in student behaviors, including implementation features. We hypothesize that intervention schools will improve their health level of the canteen, and that a healthier canteen will lead to healthier purchases. Statistical analyses will be performed using the IBM SPSS statistics version 24.0. MLwiN 2.36 software will be used to conduct the multilevel regression analyses. For all statistical analyses, a two-tailed and 5% significance level will be applied (53).

## Discussion

This study design describes how we developed and will evaluate a plan to implement guidelines to create healthier canteens in secondary schools using a systematic theory and practice-based approach. The study aims to contribute to a feasible and effective implementation of healthier school canteen policy in secondary schools. We hypothesize that schools which will receive support to implement the guidelines, will offer healthier food and beverages and that these products will be more easily accessible in the canteens compared to schools that will not receive support. In addition, we hypothesize that this will be associated with healthier purchase behavior of students in intervention schools.

Implementation of policy to limit the availability of less healthy food in schools is recommended (18) and seems effective (26). However, it also faces challenges, like conflicts with time demands for other school activities, different interests of the stakeholders (e.g., financial profit vs. healthiness), or that the implementation materials will not be used as intended. These challenges may influence the feasibility and the effectiveness of the implementation process. Although these challenges will always be present, the involvement of stakeholders during the development phase and the combination with evidence-based knowledge, frameworks and behavior change methods will result in a plan that effectively intervenes on identified challenges (28, 31). Also, the proper process evaluation will inform us about the extent of these issues. Based on all knowledge this research creates, we are able to further improve the implementation plan.

A strength of this study is the involvement of stakeholders from research, policy, and practice, which increases the support for and feasibility, usability, and impact of the intervention (9, 27, 29). As recommended, stakeholders were included in the development of the implementation plan and will be asked to share their experiences during implementation, in order to adapt the implementation tools if required (30). Acknowledged by Shea et al. it is important to have specific competencies to participate in community-engaged dissemination and implementation research (57). In the past years, the school canteen advisors of the Netherlands Nutrition Centre have already built robust partnerships with relevant stakeholders regarding healthier canteens. On the one hand, our research project will benefit from the competencies, experiences and partnerships of the advisors. On the other hand, the existing school canteen program will be improved based on the insights and results of this study.

In addition to stakeholders involvement, each school will be advised to include students in their implementation process. This because involvement of the target group facilitates implementation (58) and most students appreciate such involvement (59). We can recommend, but not prescribe how schools should involve their students, as each school has its own culture and organizational structure. The factsheet with students' needs and wishes will offer the schools insight into the opinion of their students and how they want to be involved. Our process evaluation will provide insight whether the school involved students in the implementation process.

Another strength is that we will evaluate the implementation plan using both effect and process outcomes. The effect of implementation will be measured at two levels, (i) at the student level by assessing self-reported purchase behavior and (ii) at the school level by using the Canteen Scan to measure the availability and accessibility of food and drinks in the canteen. In the process evaluation, frequently used concepts of process evaluation (dose delivered, dose received, including use, and satisfaction) will be used (43, 44). In addition, changes in factors affecting implementation will be assessed, in accordance with the demand for this knowledge (60). By this process evaluation we will be able to get some insight into which tools seem to contribute most to the implementation process. Although these conclusions should be interpreted carefully, as the tools are offered together and will probably also create a reinforcing effect.

Some limitations also need to be addressed. Measurement of the purchase behavior of students will be based on self-reporting. Alternative methods to measure purchase behavior (e.g., sales data, food measurement via observation and weighting of foods, or photographing the selected foods) have been investigated in previous studies (16, 61). However, they were considered infeasible in our study because of the time and people involved, and the differences in registration yielding incomparable sales data. Moreover, questionnaires to measure purchase behavior are commonly used in relation to consumption (18). Nevertheless, sales data and purchase behavior can be incongruent (16). The second outcome, the level of the canteen will be measured with the Canteen Scan. This tool is able to measure the level of the canteen and to give tailored feedback how to improve this level. All intervention schools will receive the feedback as an implementation tool. It can be a limitation that the same tool is used as measurement and implementation tool. However, in this study the school canteen advisors will fill out the scan, and only the intervention schools will receive the results and tailored feedback.

This study provides an example how the identified needs of stakeholders can be combined with evidence-based theory to develop an implementation plan. This study will show the impact of implementing guidelines to create healthier canteens in Dutch secondary schools, with support of the developed implementation plan, on the canteen's health level and on the purchase behavior of students. Also, the evaluation will show the appreciation, use and recommendations of the implementation tools, according to stakeholders involved in the process of creating a healthier canteen. These insights will be used to improve the existing school canteen program by supporting stakeholders to create a healthier school canteen.

## Ethics Statement

This study was carried out in accordance with the recommendations of the Medical Ethics Committee. The protocol and the procedures of informed consent in the quasi-experimental study design were approved by the VU University Medical Centre (WC2015-008 and 2015.331). All subjects gave/have to give written informed consent in accordance with the Declaration of Helsinki.

## Author Contributions

CR, EV, and JS wrote the project application. IE was the executive researcher of the studies, supported by CR, EV, and LV. IE drafted and CR, EV, LV, and JS helped to refine the manuscript. All authors approved the final manuscript.

### Conflict of Interest Statement

The authors declare that the research was conducted in the absence of any commercial or financial relationships that could be construed as a potential conflict of interest.

## Abbreviations

BCW: behavior change wheel (24)
MIDI: measurement instrument for determinants of innovation (25).
